# In-Depth Characterization of Mass Spectrometry-Based Proteomic Profiles Revealed Novel Signature Proteins Associated with Liver Metastatic Colorectal Cancers

**DOI:** 10.1155/2019/7653230

**Published:** 2019-11-04

**Authors:** Xin Ku, Yan Xu, Chunlin Cai, Yili Yang, Long Cui, Wei Yan

**Affiliations:** ^1^Shanghai Center for Systems Biomedicine, Key Laboratory of Systems Biomedicine (Ministry of Education), Shanghai Jiao Tong University, Shanghai 200240, China; ^2^Suzhou Institute of Systems Medicine, Chinese Academy of Medical Sciences, Suzhou, Jiangsu 215123, China; ^3^Colorectal Surgery Department, Xinhua Hospital, School of Medicine, Shanghai Jiao Tong University, Kongjiang Road 1665, Shanghai 200092, China

## Abstract

Liver metastasis is the most common form of metastatic colorectal cancers during the course of the disease. The global change in protein abundance in liver metastatic colorectal cancers and its role in metastasis establishment have not been comprehensively analyzed. In the present study, fresh-frozen tissue samples including normal colon/localized/liver metastatic CRCs from each recruited patient were analyzed by quantitative proteomics using a multiplexed TMT labeling strategy. Around 5000 protein groups were quantified from all samples. The proteomic profile of localized/metastatic CRCs varied greatly from that of normal colon tissues; differential proteins were mainly from extracellular regions and participate in immune activities, which is crucial for the chronic inflammation signaling pathways in the tumor microenvironment. Further statistical analysis revealed 47 proteins exhibiting statistical significance between localized and metastatic CRCs, of which FILI1P1 and PLG were identified for the first time in proteomic data, which were highly associated with liver metastasis in CRCs.

## 1. Introduction

Colorectal cancer (CRC) is the third most common cancer worldwide with an estimated incidence of 1.9 million new cases per year worldwide [1–3]. It is one of the leading causes accounting for cancer death [4, 5]. The high mortality in CRC patients is largely attributed to its late diagnosis made at advanced stage when CRC metastases had developed [6]. The 5-year survival rate of CRC patients with early localized disease was generally >50%, which decreased dramatically to less than 10% in patients with distant metastases [7, 8]. Liver metastases represented the most common form (~50%) of metastasized CRC during the course of the disease [9, 10]. The median survival time was only 5–10 months for CRC patients with liver metastases [11–13], largely due to lack of effective therapeutics [6, 14]. Although surgical removal of the metastasized tumors was feasible for some patients [15], it only increased the 5-year survival rates of these patients to ~30% [16].

Metastasis is a complicated process, during which cancer cells acquire the ability to migrate and adapt to distant microenvironments [17–20]. It has been highly demanded to identify key molecules that can provide molecular insights to address the unknown etiology of these heterogeneous, but mechanistically interesting, processes. To uncover the genetic landscape of CRC metastasis and systematically understand cellular mechanisms that favor metastasis, several genome sequencing studies were performed, which discovered a number of highly recurrent mutations in oncogenic signaling pathways [21–25]. In the proteomics field, several pioneered studies have been carried out to discover marker proteins for diagnostic purpose. However, many studies worked on formalin-fixed paraffin-embedded (FFPE) tissue samples, which have intrinsic disadvantages for proteomic analysis due to protein crosslinking issue as well as inevitable loss of proteins during the sample preparation process (multistep deparaffinization) [26]. Owing to technology advances, mass spectrometers with high resolution and sensitivity have become the method of choice for multiplexed and quantitative analysis of proteins and proteomes. In the present research, we conducted a comprehensive multiplexed proteome analysis using fresh-frozen CRC patient tissue samples. For each patient, we collected and analyzed colon/cancer/metastatic tissues to identify proteome variations not only from averaged data among different patients, as presented in a few reported researches [21, 27, 28], but also from the same genetic background. These results will deepen our insight into the molecular fingerprints of CRCs and guide the therapeutic and prognostic management in a precision manner.

## 2. Material and Methods

### 2.1. Patient Cohort

Informed consent forms were received from all patients included in this study, and all experimental work in this paper was authorized by the Xinhua Hospital Review Board and Ethical Committee.

A total number of 27 freshly frozen tissue samples from 9 patients were acquired from Xinhua Hospital, encompassing cancer tissues and the corresponding adjacent tissues of colons as well as metastatic tissues at the liver (demographic information is summarized in Table 1). The patients were not with any preradio- or chemotherapy. The histology of each recruited sample was evaluated by two pathologists using hematoxylin and eosin- (HE-) stained sections. For confusing cases and disagreement, a third pathologist would be included for further discussion.

### 2.2. Tissue Homogenization and Protein Extraction

After a careful review of all the histological information, the lysis buffer was added into each tissue sample which was precut into very small blocks. The lysis buffer contains 0.2% acid labile surfactant (ALS) in 20 mM HEPES buffer with 1X protease inhibitor (Roche, Basel, Switzerland) as reported in our previous study [29]. All the samples were placed individually in a homogenization tube with precooled ceramic beads at 4°C. After homogenization, the samples were kept half an hour on ice. Then, the lysed cells were centrifuged at 20000 g force for 0.5 h at 4°C. A standard BCA assay was applied to detect protein concentrations of all samples.

### 2.3. Protein Digestion and Peptide Purification

After lysis, the proteins were denatured by 6 M urea at room temperature for 1 h. Then, tris(2-carboxyethyl)phosphine (TCEP, 5 mM) was added to reduce the proteins at room temperature for half an hour. To alkylate the reduced proteins, iodoacetamide (IAA) was applied to each sample in 6.25 mM. The reaction mixture was incubated for 0.5 h at RT in a dark place. After that, each sample was diluted with 6 volumes of HEPES buffer (50 mM, pH = 8.2) to ensure that urea concentration is below 1 M. Sequence-modified trypsin (Promega, Madison, WI, 1 : 100 (*w*/*w*)) was added to each sample and incubated on an end-over-end shaker for 12 hours at 37°C. After digestion, the peptide mixture was quenched and acidified by phosphoric acid to pH = 2. Then, the acidic peptide mixture was loaded onto a preactivated C-18 cartridge (96-well plate, Thermo Fisher, USA). Desalting was conducted by washing 3 times with 0.1% formic acid (200 *μ*L). After that, peptides were eluted with 50% ACN and dried under vacuum with SpeedVac.

### 2.4. TMT Labeling and High-pH Fractionation

**Figure 1:**
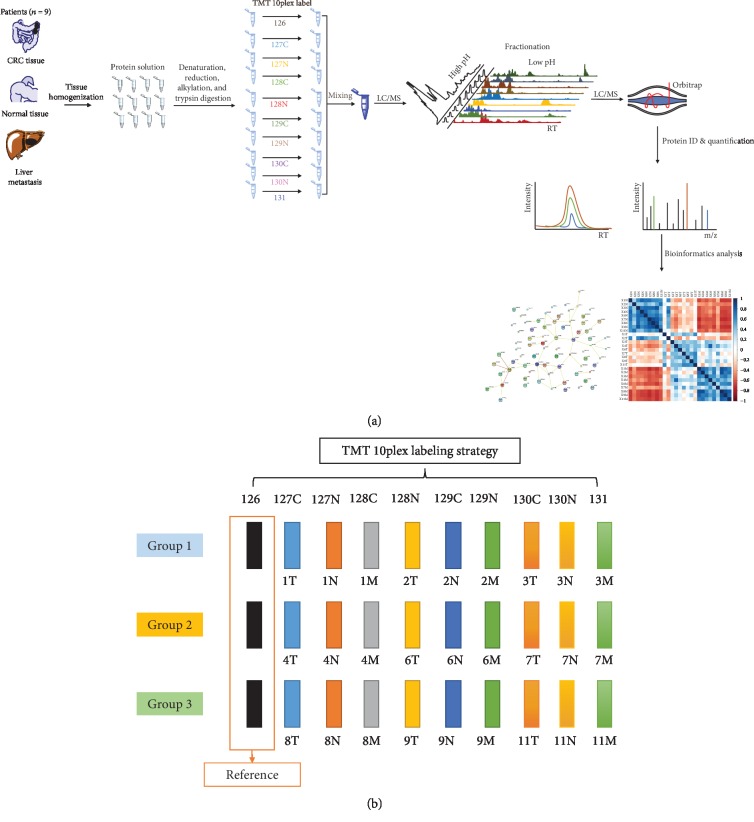
Workflow and multiplexed labeling strategy of this study. (a) Proteomic workflow of this study. (b) Labeling strategy applied in this study: peptides were labeled with TMT 10plex reagents. All tissue samples were split into three labeling batches (group 1/2/3). First, the pooled reference sample was generated by pooling aliquots of each individual sample from all patients, which was further assigned in the TMT labeling experiment as the reference channel. The 9 serial samples (T for tumor, M for metastasis, and N for normal colon) and the reference sample were together included in one TMT labeling experiment group, and there are 3 groups in total.

A common reference sample was generated by equally pooling aliquots from each peptide sample from all patients, which was applied in the designated TMT labeling experiments as the channel of reference. Serial samples within each tissue subtype (CRC/liver metastasis/normal colon) from nine patients as well as the reference channel were incorporated in each TMT 10plex labeling experiment set (see Figure 1). Dried peptides from each sample were dissolved in 200 mM HEPES buffer (pH 8.5, 1 mL for each sample). Each channel of TMT 10plex reagents (amine reactive, Thermo Fisher) was dissolved in water-free acetonitrile (ACN, 100 *μ*L). Each channel of TMT 10plex labeling reactant was mixed with the corresponding sample as described in the strategy. The mixtures were kept at 25°C for 1 h to allow the labeling reactions to complete. After that, each reaction was quenched by 5% hydroxylamine (200 *μ*L) with an incubation time of 15 min at RT. When finished, the 30 labeled samples (in 3 labeling sets) were equally mixed and separated by RPLC in basic conditions (pH = 10, 5 *μ*m, 150 × 4.6 mm, YMC, Japan) at 1 mL min^−1^. Elution buffers were as follows: basic buffer A consisted of 0.01 M NH_4_HCO_2_ in ddH_2_O and basic buffer B consisted of 0.01 M NH_4_HCO_2_ in 90% ACN (pH = 10). Finally, 100 fractions were obtained which were further concentrated into 9 fractions and dried for further mass spectrometric analysis.

### 2.5. Mass Spectrometric Analysis

Before being subjected to mass spectrometric analysis, the peptide samples were dissolved in 0.1% FA (formic acid) to reach 0.5 mg/mL. A nanoflow LC (Dionex UltiMate 3000, Thermo Fisher Scientific) was coupled to an ultra-high-resolution mass spectrometer (Orbitrap Fusion, Thermo Fisher Scientific, USA). For proteomic analysis, 1 *μ*g peptide (2 *μ*L) was separated by a self-packed analytical column (3 *μ*m particle, 75 *μ*m × 150 mm, inspire C18, Dikma, Canada) at 300 nL/min. A binary elution buffer system containing acidic buffer A (0.1% FA in ddH_2_O) as well as acidic buffer B (0.1% formic acid in ACN) was used to analyze peptides in a 62 min elution time using 7% to 35% of buffer B. The high-resolution mass spectrometer (Orbitrap Fusion) worked in a top speed, data-dependent acquisition (DDA) manner. Full-scan (MS1, mass range 350-1550 *m*/*z*) spectra were obtained at 120000 resolution with an automatic gain control of 200000 for a collection time of 100 ms in maximum. Ion signal (Si(CH_3_)_2_O)_6_ H+ at *m*/*z* 445.120025 was monitored to calibrate internal lock mass. Each selected precursor was isolated by a 1.4 *m*/*z* window, and these selected precursors were further fragmented in HCD with 32% collision energy (normalized). For MS2 spectral acquisition, the mass resolution was tuned to 60000 to achieve a clear separation of reporter ions with 6 mDa mass differences. Unassigned precursors, singly charged precursors, and higher charge-state precursors were excluded for further analysis, and recurrence of precursors was not considered within 20 s (dynamic exclusion).

### 2.6. Data Analysis

The peak lists were directly picked from acquired raw MS files and were further used to search against the UniProt protein database (*Homo sapiens*, 2016.09.16) by SEQUEST implemented in Proteome Discoverer (version 1.4, Thermo Fisher Scientific). Spectral matching was conducted using oxidation on methionine as dynamic modification and carbamidomethylation on cysteine residues as well as TMT 10plex-modified peptide N-terminus and lysine residue as static modifications. Up to two missed cleavages were tolerated while trypsin was specified as a proteolytic digesting enzyme. For precursors, the mass tolerance was allowed for 10 ppm while for fragments, the mass tolerance was restricted to 0.02 Da. The identified peptides were filtered in Proteome Discoverer at a high confidence level. A target-decoy search strategy was applied to estimate protein false discovery rate, which was filtered at 1%. The quantified intensity of the global reference served as the standard for data normalization, and only proteins identified in all three groups were considered for further analysis. Significance analysis of protein abundance variations was calculated using the pairwise two-sided Student *t*-test. The *p* values were corrected using the Benjamini-Hochberg correction when doing multiple comparisons. Further data interpretation and functional annotation were performed using DAVID, v6.8, Ingenuity Pathway Analysis (IPA), and R.

## 3. Results and Discussion

### 3.1. Overview of Patient Proteomic Profiles

As described in Figure 1(a), we collected normal colon tissue, CRC tissue, and liver metastatic tissue from each patient in the population (*n* = 9) which added up to a sample cohort of 27. Our proteomic workflow followed the general sample preparation procedure (see Figure 1), via tissue homogenization, protein alkylation, and digestion. Resulted peptides were labeled with TMT 10plex reagents. All tissue samples were split into 3 groups as three labeling batches (group 1/2/3). A sample mixture was created by pooling equally each patient sample, which served as the reference sample (see Figure 1(b) for the multiplexed labeling strategy). The mixed sample together with other nine samples of the same tissue type from each individual patient was recruited in one labeling experiment group, and there are 3 groups in total. After labeling, equal amount of each sample was mixed up and fractionated under high-pH conditions. Nine fractions were finally obtained and analyzed on nano-LC coupled with a high-resolution mass spectrometer (Orbitrap Fusion). The raw data were processed, and quantified proteins were further analyzed with bioinformatics tools.

**Figure 2:**
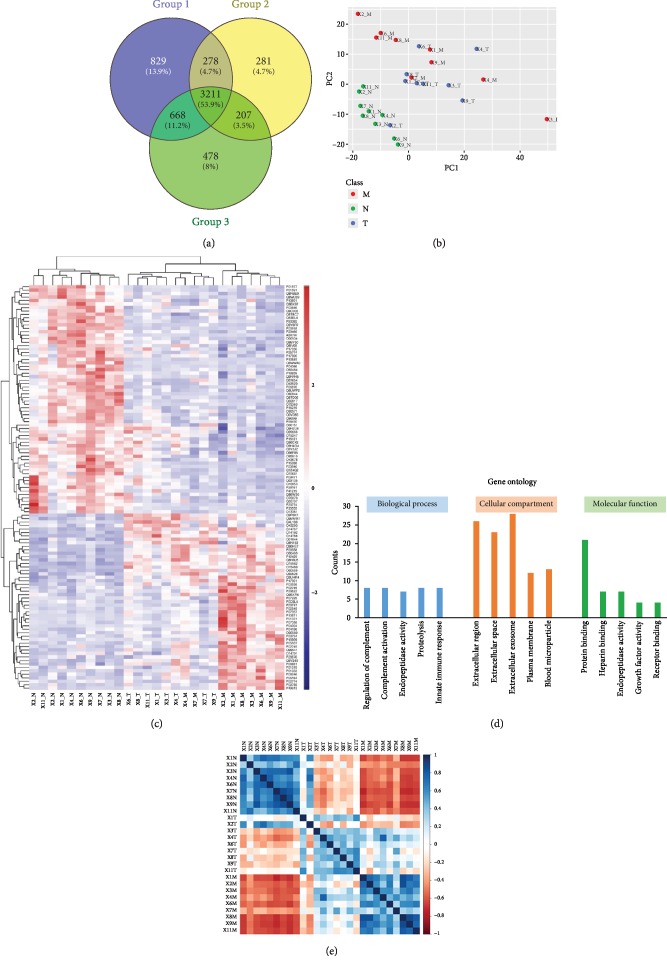
Overview of patient proteomic profiles, including normal colon (N), localized (T), and metastatic (M) CRC tissue types, as labeled at the end of each sample name. (a) Venn diagram of protein identifications from three different tissue types. (b) Principal component analysis (PCA) on the identified proteins from all samples revealed significant difference between normal colon (N) and localized (T)/metastatic (M) CRC. (c) Hierarchical cluster analysis on differentially expressed proteins among all samples. (d) Proteins with top significance (fold change > 2) were prioritized and annotated. Most of these proteins were from extracellular regions and participate in immune activities. (e) Correlation analysis of all samples. Results showed that proteomic profiles within normal tissue (N) samples were highly correlated. However, very poor correlation was found among metastatic samples which indicated the high heterogeneity of metastatic CRC.

The samples were divided into 3 groups according to their diagnostic subtypes: 9 normal colon (N), 9 CRC (T), and 9 liver metastatic tissues (M). After protein quantification and data normalization (see Material and Methods), a total of nearly 6000 proteins were quantified (Figure 2(a)), in which 3211 proteins were shared by all groups. Furthermore, we applied principal component analysis (PCA) using shared proteins and plotted the results (Figure 2(b)). It is clear that normal colon tissue represented a distinct cluster from the rest of the groups, indicating the obvious variations of proteome profiles between cancer tissues and normal tissues. Localized CRC and distant metastatic tissues represent a similar profile in terms of protein expression, resulting in an inseparable cluster in either PC1 or PC2 dimension. To explore the variation pattern among groups N, T, and M, analysis of variance (ANOVA) was utilized which led to 117 proteins with significance (*p* < 0.01). Hierarchical cluster analysis using 117 significant proteins reveals different proteome signatures among groups N and T/M (Figure 2(c)). We selected 33 most significant proteins and analyzed their functions (Figure 2(d)). In the category of cellular compartment, the majority of these proteins were identified as cell surface or exosome proteins, such as lactotransferrin (LTF), neutrophil elastase (ELANE), annexins (ANXAs), and transforming growth factor-beta-induced protein (TGFBI), which were crucial signaling molecules for cell growth and migration [30]. Most of these proteins participate in immune response and complement activation processes, probably due to the stimulation of the tumor-promoting inflammation microenvironment. Correlation analysis of these proteins among all the samples revealed that protein expression profiles were highly correlated within normal tissues (Figure 2(e)); however, the aberrant expression of proteins in the T/M group had very low correlation between individuals, indicating the high heterogeneity of cancer cells.

### 3.2. Proteome Variations between CRC, Metastasis, and Normal Colon Tissues

**Figure 3:**
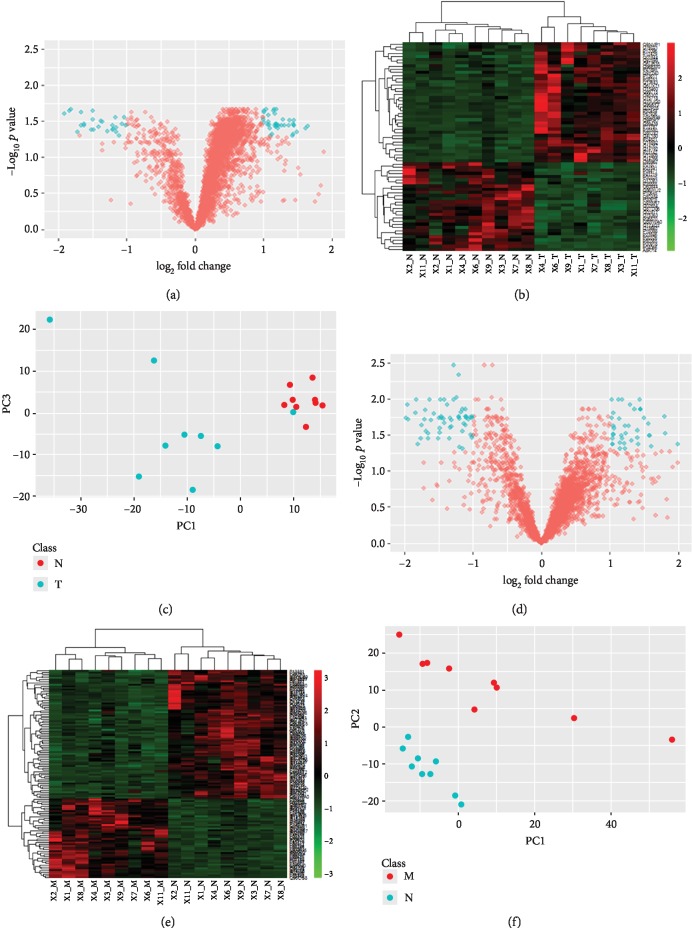
Differentially expressed proteins between different sample groups. For normal colon (N) and localized CRCs (T), volcano plot (a) revealed 66 significant proteins; these proteins were further clustered and presented in heat map (b), with 38 upregulated and 28 downregulated proteins in T (principal component analysis). (c) Using identified significant proteins showed very good separating power on PC1 dimension to distinguish normal (N) and cancer (T) tissues. Similar analysis was performed between normal colon (N) and metastatic CRC (M), with 120 significant proteins prioritized from volcano plot (d), in which 46 proteins were upregulated and 74 proteins were downregulated in M (e); very good separation between N and M on PC2 dimension was achieved using 120 significant proteins.

To further investigate tissue-specific proteome variations, we compared the proteomic profile of CRC tissue with that of normal colon tissue (T/N) as well as the proteomic profile of metastatic tissue with that of normal colon tissue (M/N). Results are summarized in Figure 3. For T/N comparison, a significant test has prioritized 66 proteins (fold change > 2, *p* < 0.05, Figure 3(a)), with 38 upregulated and 28 downregulated proteins in tumor tissues (Figure 3(b)). These proteins showed significantly different expressions between T and N, showing very good potentials to act as marker proteins/protein panel. PCA also presented that using 66 proteins, these two groups (T and N) could be well separated on PC1 dimension (Figure 3(c)). Gene ontology suggested that these proteins mainly participated in cell growth and differentiation. The number of altered proteins in terms of expression between M and N was greater than that of T/N. Under the same selection criteria (fold change > 2, *p* < 0.05), 120 proteins were shortlisted as significant proteins to characterize the main difference between groups M and N, in which 74 proteins were found to be overexpressed and 46 were found with lower expression in liver metastatic CRC tissue (Figures 3(d) and 3(e)). These proteins were mainly identified as cell surface proteins and exosomes, which control the vast majority of cellular signaling activities included in growth, invasion, and migration processes. Using the 120 proteins, groups M and N could also be separated well on PC2 dimension.

### 3.3. Investigation of Protein Expression Alterations between Local and Metastatic CRC

**Figure 4:**
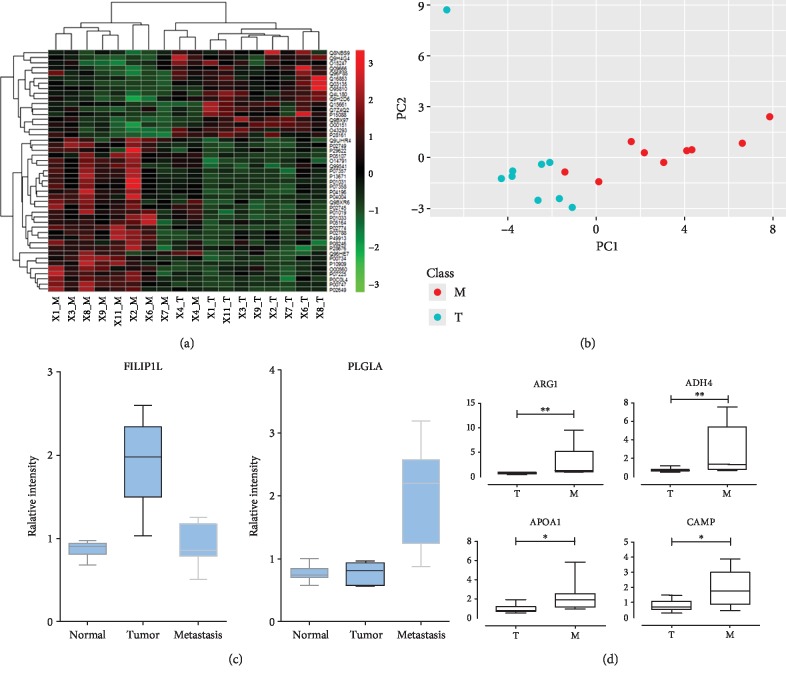
Proteins to differentiate localized (T) and metastatic (M) CRCs. Statistical analysis revealed 47 differentially expressed proteins. These proteins were presented using clustering analysis (a) and principal component analysis (b). The expression of selected significant protein candidates among different groups, e.g., normal, tumor (T), and metastasis (M), was provided in c and d, in which the *y*-axis showed the relative intensities of the proteins quantified in each tissue group.

Colorectal cancer often developed very slowly and is a highly heterogeneous disease [31]; once metastasis developed, even histopathologically similar tumors differ strikingly in terms of treatment response and survival [32]. To further study the functional roles of proteins that participated in metastasis, we compared the proteomic profile of local CRC samples (group T) with that of liver metastatic CRC samples (group M). Results are summarized in Figure 4. Statistical analysis revealed 47 proteins (*p* < 0.05) that were differentially expressed between M/T (Figure 4(a)). PCA showed a very good separation of these two groups on PC1 dimension using 47 proteins (Figure 4(b)). ANOVA (Tukey test) further prioritized two proteins with significance, FILIP1L (filamin A-interacting protein 1-like, UniProt accession: Q4L180, *p* value = 0.0096) and PLG (plasminogen, UniProt accession: P00747, *p* value = 0.03). Overexpression of FILIP1L was found to inhibit the invasion and metastasis behavior of cancer cells through the inhibition of classical WNT signaling in CRC cell lines [33–35]. The lack of FILIP1L expression (Figure 4(c)) in metastatic samples in this study could partially contribute to the metastasis of CRC cells. However, the role of this protein, especially with low expression, in normal colon cells remained to be further investigated. The PLG (plasminogen) family members were secreted proteins, which were involved in the plasminogen activation system (PAS). The expression of PAS is important in tumor spread and growth and was reported to be able to predict the outcome of human CRC [36]. It was observed in this study that significantly high expression of PLG was found in metastatic CRC samples (Figure 4(c)). We also identified a number of other proteins such as arginase-1 (ARG1) and alcohol dehydrogenase 4 (ADH4), which were overexpressed in liver metastatic CRC samples (Figure 4(d)), suggesting active roles of these proteins in liver metastasis of colorectal cancers. Further experiments to validate the functional roles of these molecules are currently ongoing.

## Figures and Tables

**Table 1:** Demographic profiles of the patient cohort.

Characteristic	Normal Colon	CRC	Liver metastasis
Patient (*n*)	9	9	9
Gender (male/female)		7/2	
Mean age (year)		55 ± 9.6	
Pathology	Colon adenocarcinoma		7
	Rectum cancer		2

## Data Availability

The data used to support the findings of this study are available from the corresponding authors upon request.

## References

[1] Siegel R. L., Miller K. D., Jemal A. (2018). Cancer statistics, 2018. *CA: A Cancer Journal for Clinicians*.

[2] Favoriti P., Carbone G., Greco M., Pirozzi F., Pirozzi R. E. M., Corcione F. (2016). Worldwide burden of colorectal cancer: a review. *Updates in Surgery*.

[3] Haggar F. A., Boushey R. P. (2009). Colorectal cancer epidemiology: incidence, mortality, survival, and risk factors. *Clinics in Colon and Rectal Surgery*.

[4] Arnold M., Sierra M. S., Laversanne M., Soerjomataram I., Jemal A., Bray F. (2017). Global patterns and trends in colorectal cancer incidence and mortality. *Gut*.

[5] Chauvin A., Boisvert F. M. (2018). Clinical proteomics in colorectal cancer, a promising tool for improving personalised medicine. *Proteomes*.

[6] Ciardiello F., Arnold D., Casali P. G. (2014). Delivering precision medicine in oncology today and in future-the promise and challenges of personalised cancer medicine: a position paper by the European Society for Medical Oncology (ESMO). *Annals of Oncology*.

[7] Creasy J. M., Sadot E., Koerkamp B. G. (2018). Actual 10-year survival after hepatic resection of colorectal liver metastases: what factors preclude cure?. *Surgery*.

[8] Tomlinson J. S., Jarnagin W. R., DeMatteo R. P. (2007). Actual 10-year survival after resection of colorectal liver metastases defines cure. *Journal of Clinical Oncology*.

[9] Chow F. C.-L., Chok K. S.-H. (2019). Colorectal liver metastases: an update on multidisciplinary approach. *World Journal of Hepatology*.

[10] van der Pool A. E. M., Damhuis R. A., IJzermans J. N. M. (2012). Trends in incidence, treatment and survival of patients with stage IV colorectal cancer: a population-based series. *Colorectal Disease*.

[11] Tosi F., Magni E., Amatu A. (2017). Effect of KRAS and BRAF mutations on survival of metastatic colorectal cancer after liver resection: a systematic review and meta-analysis. *Clinical Colorectal Cancer*.

[12] Sveen A., Løes I. M., Alagaratnam S. (2016). Intra-patient inter-metastatic genetic heterogeneity in colorectal cancer as a key determinant of survival after curative liver resection. *PLoS Genetics*.

[13] Kanas G. P., Taylor A., Primrose J. N. (2012). Survival after liver resection in metastatic colorectal cancer: review and meta-analysis of prognostic factors. *Clinical Epidemiology*.

[14] Schwartzberg L. S., Rivera F., Karthaus M. (2014). PEAK: a randomized, multicenter phase II study of panitumumab plus modified fluorouracil, leucovorin, and oxaliplatin (mFOLFOX6) or bevacizumab plus mFOLFOX6 in patients with previously untreated, unresectable, wild-type KRAS exon 2 metastatic colorectal cancer. *Journal of Clinical Oncology*.

[15] Van Cutsem E., Cervantes A., Adam R. (2016). ESMO consensus guidelines for the management of patients with metastatic colorectal cancer. *Annals of Oncology*.

[16] Jones R. P., Jackson R., Dunne D. F. J. (2012). Systematic review and meta-analysis of follow-up after hepatectomy for colorectal liver metastases. *The British Journal of Surgery*.

[17] Yuge R., Kitadai Y., Shinagawa K. (2015). mTOR and PDGF pathway blockade inhibits liver metastasis of colorectal cancer by modulating the tumor microenvironment. *The American Journal of Pathology*.

[18] Giakoustidis A., Mudan S., Hagemann T. (2015). Tumour microenvironment: overview with an emphasis on the colorectal liver metastasis pathway. *Cancer Microenvironment*.

[19] Zou Y. F., Cai Z. R., Chen Y. F. (2013). Comparison of local immune microenvironment between liver-metastasis colorectal cancer and non-liver-metastasis colorectal cancer. *Zhonghua Wei Chang Wai Ke Za Zhi*.

[20] Eveno C., Pocard M. (2012). VEGF levels and the angiogenic potential of the microenvironment can affect surgical strategy for colorectal liver metastasis. *Cell Adhesion & Migration*.

[21] Lee P. Y., Chin S. F., Low T. Y., Jamal R. (2018). Probing the colorectal cancer proteome for biomarkers: current status and perspectives. *Journal of Proteomics*.

[22] The Cancer Genome Atlas Network (2012). Comprehensive molecular characterization of human colon and rectal cancer. *Nature*.

[23] Krausova M., Korinek V. (2014). Wnt signaling in adult intestinal stem cells and cancer. *Cellular Signalling*.

[24] Kim T. M., Lee S. H., Chung Y. J. (2013). Clinical applications of next-generation sequencing in colorectal cancers. *World Journal of Gastroenterology*.

[25] Spier I., Horpaopan S., Vogt S. (2012). Deep intronic APC mutations explain a substantial proportion of patients with familial or early-onset adenomatous polyposis. *Human Mutation*.

[26] Thompson S. M., Craven R. A., Nirmalan N. J., Harnden P., Selby P. J., Banks R. E. (2013). Impact of pre-analytical factors on the proteomic analysis of formalin-fixed paraffin-embedded tissue. *Proteomics Clinical Applications*.

[27] Coghlin C., Murray G. I. (2015). Biomarkers of colorectal cancer: recent advances and future challenges. *Proteomics Clinical Applications*.

[28] Peltier J., Roperch J. P., Audebert S., Borg J. P., Camoin L. (2016). Quantitative proteomic analysis exploring progression of colorectal cancer: modulation of the serpin family. *Journal of Proteomics*.

[29] Sun Q., Ku X., Xu N., Zhang X., Yan W., Fang W. (2018). Investigation of an optimal lysis method for the study of thymus and thymoma by mass spectrometry-based proteomics. *Translational Cancer Research*.

[30] Maji S., Chaudhary P., Akopova I. (2017). Exosomal annexin II promotes angiogenesis and breast cancer metastasis. *Molecular Cancer Research*.

[31] Brenner H., Stock C., Hoffmeister M. (2014). Effect of screening sigmoidoscopy and screening colonoscopy on colorectal cancer incidence and mortality: systematic review and meta-analysis of randomised controlled trials and observational studies. *BMJ*.

[32] Akkad J., Bochum S., Martens U. M. (2015). Personalized treatment for colorectal cancer: novel developments and putative therapeutic strategies. *Langenbeck's Archives of Surgery*.

[33] Kwon M., Kim J. H., Rybak Y. (2016). Reduced expression of FILIP1L, a novel WNT pathway inhibitor, is associated with poor survival, progression and chemoresistance in ovarian cancer. *Oncotarget*.

[34] Kwon M., Libutti S. K. (2014). Filamin A interacting protein 1-like as a therapeutic target in cancer. *Expert Opinion on Therapeutic Targets*.

[35] Kwon M., Lee S. J., Wang Y. (2014). Filamin A interacting protein 1-like inhibits WNT signaling and MMP expression to suppress cancer cell invasion and metastasis. *International Journal of Cancer*.

[36] Seetoo D. Q., Crowe P. J., Russell P. J., Yang J. L. (2003). Quantitative expression of protein markers of plasminogen activation system in prognosis of colorectal cancer. *Journal of Surgical Oncology*.

